# Inhibition of X-linked inhibitor of apoptosis protein suppresses tumorigenesis and enhances chemosensitivity in anaplastic thyroid carcinoma

**DOI:** 10.18632/oncotarget.21320

**Published:** 2017-09-28

**Authors:** Yao Liu, Bing Zhang, Tiefeng Shi, Huadong Qin

**Affiliations:** ^1^ The Fourth Department of General Surgery, The Second Affiliated Hospital of Harbin Medical University, Harbin, Heilongjiang, People's Republic of China

**Keywords:** anaplastic thyroid carcinoma (ATC), X-linked inhibitor of apoptosis protein (XIAP), proliferation, migration, invasion

## Abstract

Anaplastic thyroid carcinoma (ATC) is one of the most lethal carcinoma with a poor prognosis; however, molecular mechanisms underlying the aggressiveness of ATC remain unclear. Our goal was to examine the expression of X-linked inhibitor of apoptosis protein (XIAP) in ATC, as well as its role in ATC tumorigenesis. This is a retrospective study of ATC patients from the Second Affiliated Hospital of Harbin Medical University during June 2003 to October 2013. The expression of XIAP in tumor specimens of ATC patients was examined by immunohistochemical staining. The roles of XIAP in proliferation, migration, invasion, and chemoresistance were investigated by shRNA mediated-knockdown of XIAP in human ATC cell lines. The effect of XIAP on tumorigenesis was evaluated using a xenograft tumor model with nude mice. XIAP expression was significantly higher in the invasive area of ATC samples, whereas XIAP expression was negative in either normal thyroid follicular epithelial cells or the differentiated papillary thyroid carcinoma. XIAP-depleted ATC cells showed a remarkable decrease in the proliferation, migration, and invasion compared with the scramble group. Knockdown of XIAP expression significantly enhanced the chemosensitivity of WRO and SW1736 cells to docetaxel or taxane. Moreover, knockdown of XIAP significantly suppressed ATC tumorigenesis *in vivo*. XIAP is highly expressed in ATC cells and tumors. XIAP play important roles in tumor behaviors and chemosensitivity of ATC cells. XIAP may function in ATC aggressiveness and may serve as a potential therapeutic target for ATC treatment.

## INTRODUCTION

Anaplastic thyroid carcinoma (ATC) is a rare and particularly highly aggressive subtype of thyroid malignancy and induces up to 14%–39% of thyroid cancer-related deaths [1]. Current treatment strategies fail sometimes due to therapeutic resistance; therefore more effective treatment strategies for ATC are still urgently needed. The X-linked inhibitor of apoptosis protein (XIAP) is one of the inhibitors of apoptosis (IAP) family members, which mainly functions as a potent suppressor involved in apoptosis [2]. Aberrant expression of XIAP was found in a variety of human cancers, including esophageal carcinoma, acute leukemia, non-small cell human lung cancer, ovarian carcinoma, bladder cancer, and several other carcinomas [3–8]. Increased XIAP expression has been shown to correlate with chemoresistance of cancer cells to drugs and radiotherapy [9, 10]. Whereas, decreased expression of XIAP sensitizes drug-resistance of cancer cells to apoptosis [11, 12]. Therefore, XIAP may serve as a potential diagnostic and therapeutic target for antineoplastic therapy. However, the role of XAIP in ATC is unclear. In this study, we investigated the expression pattern as well as the function of XIAP in ATC. Moreover, we evaluated the impact of XIAP depletion on chemoresistance of ATC cells. We found that XIAP expression is upregulated in ATC cells and tissues. XIAP has a tumor-promoting role in ATC and depletion of XIAP increases the chemosensitivity of ATC cells.

## RESULTS

### XIAP is abundantly expressed in ATC

**Figure 1:**
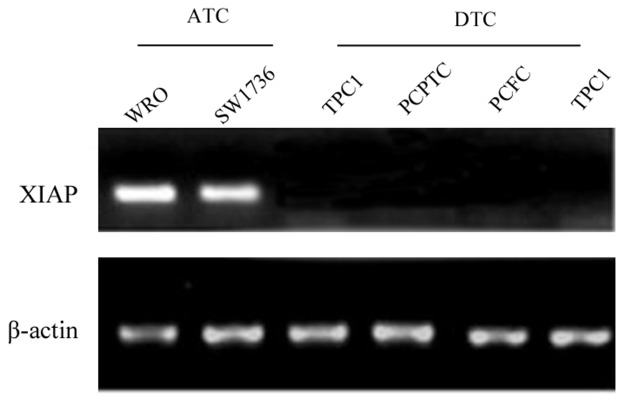
XIAP was abundantly expressed in the ATC cell lines WRO and SW1736, whereas, no expression was found in DTC cell lines

**Figure 2:**
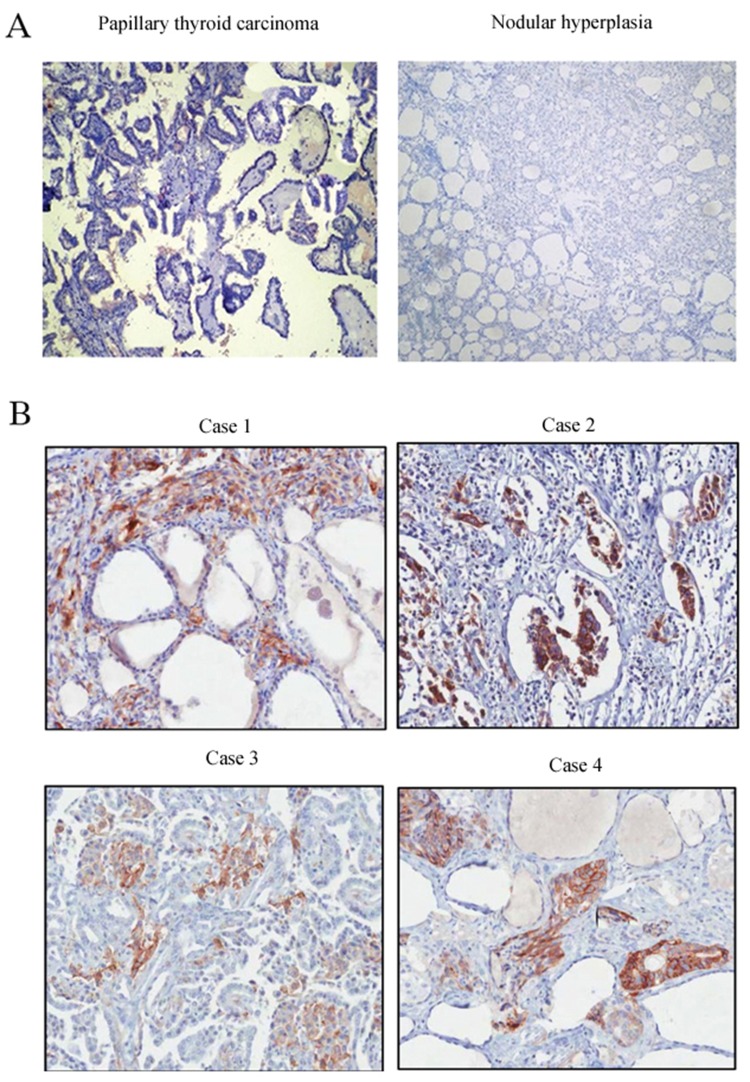
Immunohistochemical staining of XIAP in different tissues **(A)**, Nodular hyperplasia and PTC tissues; **(B)**, ATC tissues (×200).

*In vitro* experiments showed that XIAP was expressed in the ATC cell lines WRO and SW1736, whereas, no expression of XIAP was detected in the primary cultured follicular cells or the differentiated thyroid cancer cell lines (Figure 1), indicating that XIAP may serve as a specific biomarker of ATC tumors. Next, we determined XIAP expression in ATC patients and patients with well-differentiated PTCs and nodular hyperplasia. Clinicopathologic features of patients are shown in Table 1. The median age of 16 ATC patients was 57 (range, 38 to 81) years old, and 62.5% were male. Most patients were reported as stage IVB disease (n = 13, 81.2%). Of the 16 samples, 12 (75%) showed pure ATC, whereas the remaining four (25%) showed evidence of concomitant DTC in the same pathologic samples. Immunohistochemical analysis showed negative XIAP expression in well-differentiated PTCs and nodular hyperplasia (Figure 2A), while XIAP was positively expressed in most tumor tissues of ATC patients (n = 12), with abundant staining in membranous. Four representative images demonstrated strong XIAP expression was displayed in the invasive areas of ATC tumors (Figure 2B). Taken together, these data suggested that XIAP expression is highly expressed in ATC tumors. These data indicated that XIAP expression varies in different ATC cases which may not be correlated with tumor metastasis and invasion.

**Table 1:** Clinicopathologic features of XIAP high vs. low expression in ATC patients

	High PD-L1 Expression[N (%)]	Low PD-L1 Expression[N (%)]	Total Patients[N (%)]
Sex			
Male	7 (58.3)	3 (75)	10 (62.5)
Female	5 (41.7)	1 (25)	6 (37.5)
Median age, y	61 (range, 49–73)	55 (range, 38–81)	57 (range, 38–81)
Histology			
Pure ATC	3 (60)	9 (81.8)	12 (75)
With DTC	2 (40)	2 (11.2)	4 (25)
Stage (AJCC 7th ed)			
IVA	1 (14.3)	0 (0)	2(12.5)
IVB	5 (71.4)	8 (80)	13 (81.2)
IVC	1 (14.3)	2 (20)	1 (6.3)
Surgery			
Yes	6 (100)	8 (80)	14 (87.5)
No	0	2 (20)	2 (12.5)
Resection status			
R0	2 (25)	1 (12.5)	3 (18.8)
R1	5 (62.5)	4 (50)	9 (56.2)
R2	1 (12.5)	3 (37.5)	4 (25)
Chemoradiation	6 (100)	10 (100)	16 (100)
Chemotherapy			
Cisplatin	0	1 (12.5)	1 (6.2)
Doxorubicin	1 (12.5)	2 (25)	3 (18.8)
Docetaxel plus doxorubicin	5 (62.5)	4 (50)	9 (56.2)
Carboplatin plus taxane	2 (25)	1 (12.5)	3(18.8)

### Roles of XIAP in proliferation, migration, and invasion of ATC cells

**Figure 3:**
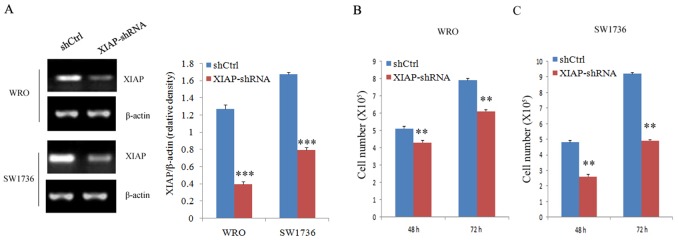
Cell proliferation evaluation in ATC cell lines after XIAP shRNA transfection **(A)**, Western blot analysis of XIAP expression in WRO and SW1736 cells transfected with XIAP-shRNA; **(B)** and **(C)**, Survival of WRO and SW1736 cells transfected with XIAP shRNA transfection. All experiments were carried out in triplicate. Data were expressed as mean±SD, ^**^P < 0.01, ^***^P < 0.001 vs. shCtrl group.

**Figure 4:**
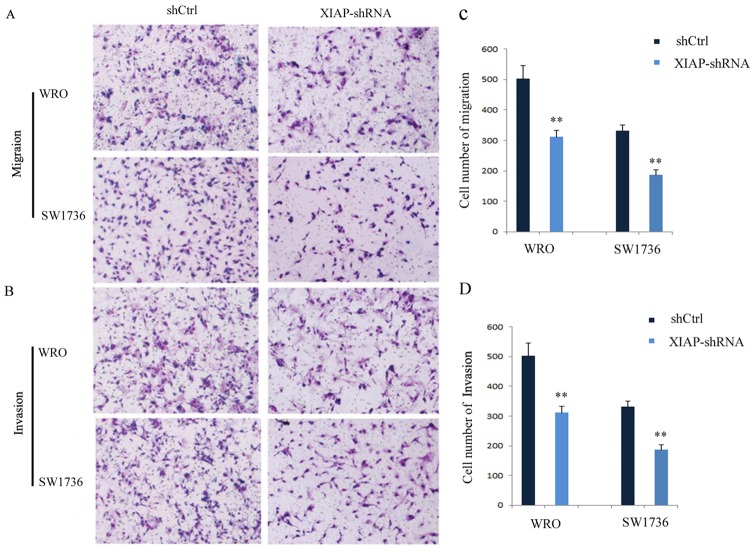
Knockdown of XIAP suppresses the migration and invasion of ATC cells **(A)** Migration of WRO and SW1736 cells; **(B)** Invasion of WRO and SW1736 cells; **(C)** Quantification of migration of WRO and SW1736 cells; **(D)** Quantification of invasion of WRO and SW1736 cells. All experiments were carried out in triplicate. Data were expressed as mean±SD, ^**^P < 0.01 vs. shCtrl group.

To examine whether XIAP plays an important role in ATC proliferation, migration, and invasion, stable XIAP knockdown FRO or 8505C cell clones were generated by lentiviral-mediated transduction with XIAP-shRNA (Figure 3A). As shown in Figure 3B, the proliferation rates of WRO cells at 48 hours (14.8% ± 3.1%) and 72 hours (23.4% ± 2.7%; P < 0.01) were significantly decreased in XIAP-shRNA group compared with those in the scramble group. Also, the proliferation rates of SW1736 cells at 48 hours (44.1% ± 7.6%) and 72 hours (45.5% ± 4.3%; P < 0.01) were significantly decreased in XIAP-shRNA group compared with those in the scramble group (Figure 3C). Transwell assay showed that the migration (43.7% ± 6.4%) and invasion (42.2%±3.8%) capabilities of WRO cells were remarkably decreased in XIAP-shRNA group compared with those in the scramble group (Figure 4A and 4B; P < 0.01). The migration (40.6% ± 5.3%) and invasion (35.9% ± 10.5%) capabilities of SW1736 cells were also decreased significantly (Figure 4C and 4D; P < 0.01). Together, these results suggested that XIAP plays a critical role in the proliferation, migration, and invasion of ATC cells.

### Association of XIAP expression with chemoresistance in ATC

**Figure 5:**
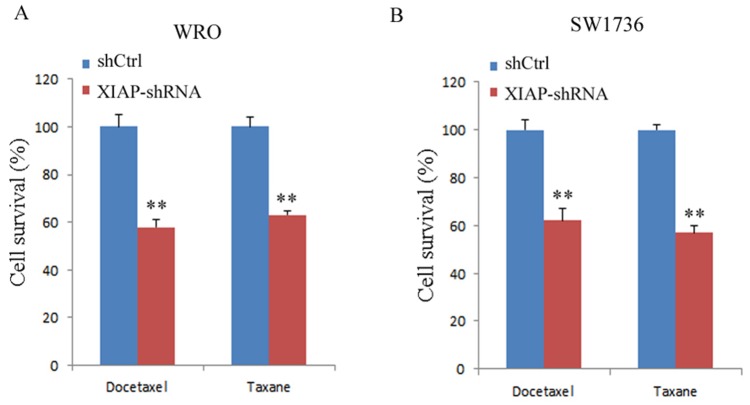
XIAP depletion enhanced chemosensitivity of WRO and SW1736 cell lines to docetaxel or taxane **(A)**, Viability of WRO cells was determined by trypan blue exclusion; **(B)**, Viability of SW1736 cells was determined by trypan blue exclusion. All experiments were carried out in triplicate. Data were expressed as mean±SD, ^**^P < 0.01 vs. shCtrl group.

Next, we examined whether XIAP expression was correlated with the chemosensitivity of SW1736 cells to docetaxel or taxane. Our data showed that the cell viability decreased significantly in XIAP-depleted WRO cells after treatment with docetaxel (40.4% ± 6.3%) or taxane (35.7% ± 5.1%) compared with control cells (P < 0.01, Figure 5A). Similarly, depletion of XIAP significantly decreased the cell growth rate of SW1736 cells after docetaxel (35.8% ± 3.6%) or taxane (42.3% ± 5.8%) treatment (P < 0.01, Figure 5B). Our results supported the hypothesis that XIAP expression is associated with chemoresistance in ATC cells.

### Impact of XIAP on tumor formation in an ATC xenograft mouse model

**Figure 6:**
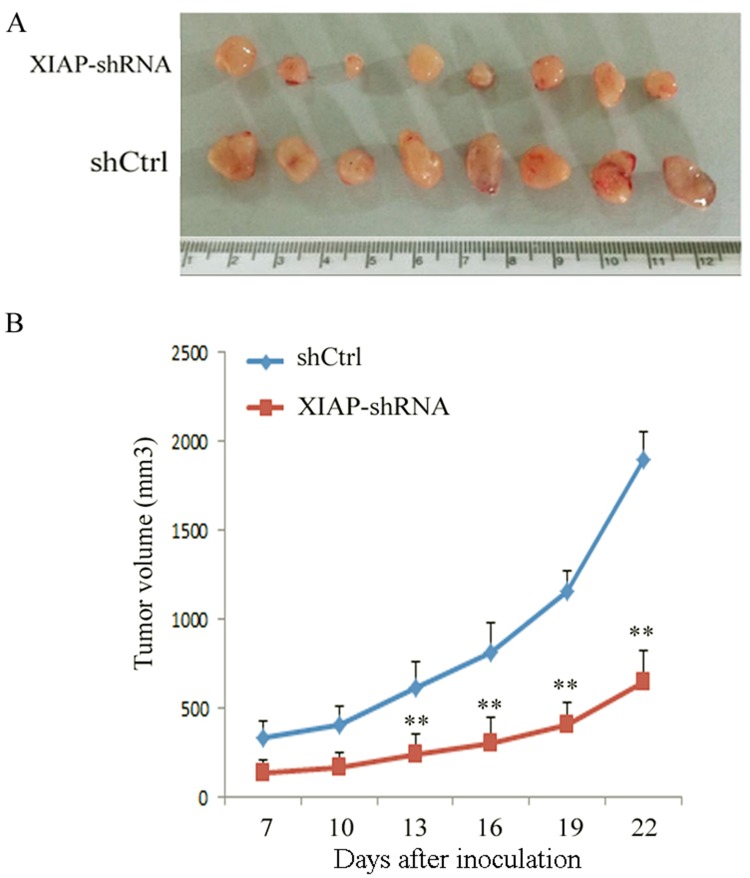
Knockdown of XIAP decreased the tumor growth of tumor-bearing nude mice in an ATC xenograft model **(A)**, WRO cells transfected with XIAP shRNA or shCtrl were inoculated into nude mice, and photographs of the tumors at autopsy are presented; **(B)**, The tumor growth was monitored in XIAP shRNA or shCtrl group during the indicated period. Data were expressed as mean±SD, ^**^P < 0.01 vs. shCtrl group.

Finally, we evaluated the impact of XIAP on tumor formation *in vivo* using an ATC xenograft mouse model. XIAP-depleted ATC cells were injected into the right flank of BALB/c nude mice. As compared with the control shRNA group, a significant decrease of tumor growth was observed in mouse xenografts with the XIAP-depleted ATC cells (Figure 6A and 6B). Twenty-two days after injection, we found a remarkable tumor volume decrease in XIAP-shRNA mice compared with the control shRNA mice (651.2±170.5 mm3 vs. 1,894.6±157.1 mm3, P< 0.01). These results indicated that XIAP promotes tumor growth and formation of ATC.

## DISCUSSION

Thyroid cancer (TC) has been recognized as a common endrocrine malignancy, the majority of which are well-differentiated TC. PTC and FTC are the top two frequent subtypes, representing 80–84% and 6–10% of all thyroid carcinomas, respectively [13]. According to the 2012 GLOBOCAN data, the incidence of TC worldwide has reached above 1% of all oncological diseases [14]. XIAP, a member of IAP protein family, has been reported to be upregulated in various types of cancers and is involved in tumor progression [15]. It has been well-documented that XIAP has potential inhibitory function in apoptosis [16–19]. The role of XIAP in breast cancer and clear-cell ovarian cancer has been elucidated by RNA interference method [20, 21]. The associations between XIAP expression and aggressive clinical behaviors and tumor progression have been found in many literatures [22]. Chen *et al*. reported that XIAP expression is correlated with chemosensitivities of cells to drugs, including methotrexate [23]. Increasing researches have focused on XIAP inhibition to develop new therapies for cancer prevention and treatment [24]. To date, a number of signaling pathways have been identified to be involved in regulating XIAP expression, including p53, NF-κB, c-Jun/AP-1, and PI3K dependent signaling pathways.

In this study, we showed high XIAP expression in ATC cell lines, but no XIAP expression in the primary cultured follicular cells or the differentiated thyroid cancer cell lines, which was in line with the aberrant XIAP expression found in the invasive area of ATC samples. These data indicated that ATC tumors and cell lines have specific expression pattern of XIAP compared with thyroid cells, indicating an important role of XIAP in ATC. To further explore the role of XIAP in ATC, we knocked down XIAP in ATC cells. We found that XIAP-depleted ATC cells had decreased cell viability and migration and invasion capability. Consistently, a significant decrease in tumor growth was observed in XIAP-depleted ATC xenografts in nude mice. These data suggested a tumor-suppressive role of XIAP in ATC.

A recent study showed that single chemotherapeutic drug exposure to ATC cells has antitumor activity [25]. However, combination treatment of these drugs shows no statically significant synergistic antitumor activity, indicating that combination therapy may have limited effectiveness for overcoming chemoresistance of ATC. We wondered whether knockdown of XIAP would enhance the chemoresistance of ATC cells. Interestingly, we found that knockdown significantly increased the sensitivity of ATC cells to docetaxel or taxane. Together, our results imply that XIAP is critical for tumor progression and chemoresistance of ATC.

## CONCLUSION

In conclusion, our results showed that XIAP is highly expressed in ATC tumors and plays a vital role in proliferation, migration, invasion, and chemosensitivity of ATC cells *in vitro*. Additionally, our data also showed that XIAP knockdown ATC-xenografted nude mice have inhibited tumor growth. Our results indicate combined targeted therapy of XIAP with chemotherapy may be a novel treatment strategy for ATC treatment.

## MATERIALS AND METHODS

### Patients and tissues

A total of 16 ATC patients who underwent thyroid surgery during June 2003 to October 2013 at the Fourth Department of General Surgery, The Second Affiliated Hospital of Harbin Medical University were retrospectively selected. All tumor specimens were histopathologically examined by three experienced pathologist. Written consents were obtained from the patients undergoing the surgery. All the research protocols were approved by the Ethics Committee of the Second Affiliated Hospital of Harbin Medical University.

### Cell culture

Human ATC cell lines WRO (a kind gift from Dr. Pan, Guangdong Medical University) and SW1736 were maintained in RPMI-1640 (Invitrogen) with 5% FBS (Hyclone). Human papillary thyroid carcinoma (PTC) cell lines FB-2 and TPC-1 (kind gifts from Dr. Yao, Affiliated Zhongshan Hospital of Dalian University) were cultured in Dulbecco's Modified Eagle’sMedium (DMEM, Invitrogen) with 10% FBS. Primary PTC and follicular thyroid carcinoma (FTC) cells were cultured in RPMI-1640 medium supplemented with 10% fetal bovine serum, 0.1 mM nonessential mino acids, 1 mM sodium-pyruvate, and 100 U/100 μg/ml of penicillin/streptomycin. Human ovary adenocarcinoma cells SPEC-2 were cultured in McCoy's 5A (Invitrogen) with 10% FBS. All cells were maintained in 5% CO2 at 37°C.

### Hematoxylin-eosin staining

Formalin-fixed tumor specimens were embedded in paraffin and stained with hematoxylin-eosin for conventional morphological evaluation under a light microscope.

### Immunohistochemical staining

Immunohistochemical analysis of XIAP was performed according to the standard procedures. Briefly, tumor tissue slides (3-μm thick) were deparaffinized in xylene and rehydrated in graded alcohol. After antigen retrieval, tissue sections were treated with 3% hydrogen peroxide for 10 minutes to block endogenous peroxidases. Sections were incubated at 4°C overnight with rabbit anti-XIAP mAb (1:200; cat: IMG-5770, Novus Biologicals) in a humid chamber. Mouse IgG1 was used as a negative control, and peripheral nerve bundles were used as an internal positive control. Scoring was semiquantitatively assessed by the cell cytoplasm staining pattern of tumor or nontumorous tissues as described previously [26]: The staining intensity was scored as 0, absent staining; 1, weak staining; 2, moderate staining; and 3, strong staining. The staining extent (percentage of positive cells) was scored as 0, absent staining; 0.1, light brown staining; 0.5, intermediate staining; and 1, dark brown staining. A final score ranging from 0 to 3 was calculated by staining extent multiplied by staining intensity. Samples were considered positive if the final score was ≥3. XIAP high vs. low expression was determined with respect to the median percentage staining for each group.

### Lentivirus packaging

Four different short hairpin RNA (shRNA) nucleotide sequences were inserted into the pFH1UGW-GFP vector (Hollybio, Shanghai, China) and were confirmed by DNA sequencing. A random shRNA sequence (shCtrl) (5’-CCGGCCAAGTTAATTCGGCCCGCGGC TCTCGAGAAGCGTTACTTAAGG-3’) was used as a control (Hollybio, Shanghai, China). Lentiviruses were generated via the transfection of 293T cells using Lipofectamine (Invitrogen) at 70-80% confluence according to the manufacturer's instructions. After 12 hours, the virus generated in the supernatants was filtered through a 45-μm filter, and centrifuged (4, 000 g at 4°C) for 15 min. The concentrated lentiviruses were then added into SW1736 and WRO cells with 5 μg/mL polybrene. After incubation for 24 h, the medium was replaced with fresh media supplemented with 1.2 μg/mL puromycin. Puromycin resistant clones were selected after culture for 1 week in the presence of puromycin. To confirm the knockdown efficiency of XIAP, four different shRNA clones were evaluated by reverse transcriptase PCR and Western blot. Among them, a highly functional XIAP-targeting shRNA (5’- GGCTCACCTATCGAGTCGTTTCATAACATGGTACCTGGGCAAGGATAC-3’) was chosen for further experiments.

### Cell migration, invasion, and cell survival assays

Cells (1×10^5^cells/mL) were seeded in 6-well plates in 2 mL of media containing 2% FBS. After 72 hours, viable cells were counted in a cell counter (Innovatis AG). Transwell migration assays were performed with Transwell insert chambers (Corning, New York, NY). Briefly, a density of 1×10^6^ cells were seeded in the upper chambers of Transwell in RPMI-1640 media. After incubation for 24 h, cells were fixed with methanol for 5 min and stained with hematoxylin and eosin for 5 min. Cells on the upper surface of the filter was then removed through wiping with a cotton swab. The migrated cells were examined by imaging analysis. Cells in eight random microscopic fields with an optical microscope (200× magnification) were counted per well. Transwell invasion assays were performed using the same protocol as the migration assay, except that the transwell filters were coated was pre-coated with 100 μl of 2% Matrigel (BD Biosciences, San Jose, CA) before cell seeding. For cell survival assay, cells were seeded in 6-well plates in 2 mL of RPMI-1640 containing 5% FBS. After incubation for 48 hours, cell viability was determined by Trypan Blue exclusion. The assay was performed in triplicate.

### Western blot analysis

Cells were lysed and proteins were separated by 10% SDS-PAGE and transferred to polyvinylidene fluoride (PVDF) membranes (Chemicon). The membranes were blocked with 5% skimmed milk for 1 hour at room temperature. Then, the membranes were incubated with primary antibodies against XIAP (rabbit polyclonal antibody, cat: ab86229, Abcam) or β-actin (mouse monoclonal antibody, internal control, cat: ab8226, Abcam) diluted 1:1500 overnight at 4°C followed by horseradish peroxidase (HRP)-conjugated secondary antibodies (Santa Cruz Biotechnology, CA) diluted 1:2000 for 1 hour at room temperature. Band densities were quantified with the Image J program.

### ATC xenograft assay in a nude mouse model

BALB/c nude mice (4-6 weeks old, 18 ± 4 g) were purchased from Shanghai Laboratory Animal Center, Chinese Academy of Sciences. Mice were randomized into two groups (n=8) and were housed under SPF conditions. XIAP-shRNA or Ctrl-shRNA transfected WRO cells were subcutaneously injected into the right flank of each mouse. Tumor volumes were calculated using the formula V (mm3) = ½(AxB2) as described previously [27]. The animal protocol was approved by the Animal Care and Use Committee of the Second Affiliated Hospital of Harbin Medical University.

### Statistical analysis

Data were analyzed by SPSS software version 22.0 (SPSS Inc., Chicago, IL). The data were expressed as mean ± SD. Statistical evaluation was performed using Mann-Whitney U Test. P-value less than 0.05 was considered statistically significant.

## Abbreviations

ATC: Anaplastic thyroid carcinoma
XIAP: X-linked inhibitor of apoptosis protein
PTC: papillary thyroid carcinoma
FTC: follicular thyroid carcinoma
